# Factors influencing the length of hospital stay of patients with anorexia nervosa – results of a prospective multi-center study

**DOI:** 10.1186/s12913-017-2800-4

**Published:** 2018-01-15

**Authors:** D. Kästner, B. Löwe, A. Weigel, B. Osen, U. Voderholzer, A. Gumz

**Affiliations:** 1 Department of Psychosomatic Medicine and Psychotherapy, University Medical Center, Hamburg-Eppendorf & Schön Clinic Hamburg Eilbek, Martinistr. 52, W37, 20246 Hamburg, Germany; 2 Schön Clinic Bad Bramstedt, Bad Bramstedt, Germany; 3 Schön Clinic Roseneck, Prien, Germany; 4 Clinic for Psychiatry and Psychotherapy, University of Freiburg, Freiburg, Germany

**Keywords:** Anorexia nervosa, Inpatient, Hospitalization, Length of stay, Predictors

## Abstract

**Background:**

The length of stay (LOS) strongly influences anorexia nervosa (AN) inpatient weight outcomes. Hence, understanding the predictors of LOS is highly relevant. However, the existing evidence is inconsistent and to draw conclusions, additional evidence is required.

**Methods:**

We conducted a prospective, multi-center study including adult female inpatients with AN. Using stepwise linear regression, the following demographic and clinical variables were examined as potential predictors for LOS: admission BMI, AN-subtype, age, age of onset, living situation, partnership status, education, previous hospitalization, self-rated depression, anxiety and somatic symptoms (PHQ-9, PHQ-15, GAD-7), self-rated therapy motivation (FEVER) and eating disorder psychopathology (EDI-2 subscale scores).

**Results:**

The average LOS of the sample (*n* = 176) was 11.8 weeks (SD = 5.2). Longer LOS was associated with lower admission BMI (ß = −1.66; *p* < .001), purging AN-subtype (ß = 1.91; *p* = .013) and higher EDI-2 asceticism (ß = 0.12; *p* = .030). Furthermore, differences between treatment sites were evident.

**Conclusions:**

BMI at admission and AN-subtype are routinely assessed variables, which are robust and clinically meaningful predictors of LOS. Health care policies might consider these variables. In light of the differences between treatment sites future research on geographical variations in mental health care seems recommended.

## Background

Anorexia nervosa (AN) is a severe mental disorder characterized by self-induced underweight (body mass index, BMI <17.5) and associated with serious medical complications, impairments in psychosocial functioning and psychiatric comorbidities [1–3]. Chronic courses are common and mortality rates are high [4, 5]. Due to the severity of the disorder a considerable proportion of individuals who are diagnosed with AN receives inpatient therapy [6]. This finding holds true especially within the European health care services [1, 7, 8]. One of the principal goals of inpatient treatment for patients with AN is the normalization of body weight. The importance of the short-term weight-related outcomes (e.g., discharge weight, inpatient BMI gain) for the subsequent illness course (e.g., readmission) has been demonstrated by numerous studies [9–12]. As one would expect, the length of the hospital stay (LOS) has been found to have the strongest influence on inpatient weight outcomes [13, 14]. Moreover, a longer length of the first hospital admission has been found to be associated with decreased mortality in the long-term [15].

Despite these findings, there is no evidence-based consensus on the optimal LOS of a certain AN patient in inpatient treatment and the recommended LOS therefore varies greatly between specialized institutions [16]. Over time, a trend towards shorter inpatient treatment durations has been observed due to increased economic pressure [17–19]. To date, decisions about the highly outcome-related LOS are made on the basis of clinical judgment and in light of financial constraints [20]. Therefore, it is highly relevant to investigate factors that influence a decision towards a longer or shorter inpatient stay and to evaluate if such factors are in line with clinical theory. Such evidence could provide a basis for a more standardized and need-oriented determination of inpatient treatment durations. Ultimately, such findings could serve to refine reimbursement systems.

So far, it is known that the diagnosis of an eating disorder is associated with longer hospital stays than other psychological disorders [21]. The existing evidence pertaining to the predictors of LOS within inpatients with AN is summarized in Table 1. While the evidence base is fairly large, few predictors have been replicated consistently. To draw conclusions for current health care policies, additional evidence is required.

**Table 1:** Evidence on predictors of LOS in inpatients with AN

DV	Positive predictors	Negative predictors	Non-influential variables
LOS	age [36] previous admission [34] comorbid mental disorder [35, 45]^a^duration of illness [45]^a^ NG tube feeding [45, 46]^a^involuntary admission [46]^a^	t0 BMI [34] [35]^a^minimum weight after onset [36] adherence to therapeutic contract [45]^a^	treatment site, area of residence, menstrual status, education, abnormal white cell count [34] age at onset, t0 weight, duration of illness, previous admissions [36] previous admission, age at onset, t0 BMI, duration of untreated illness, distance from hospital, socioeconomic level, menstrual status, education [45]^a^ age, gender, number of comorbidities, time (2005-2009) [35]^a^

*DV* Dependent variable, *t0* admission, *BMI* Body mass index. ^a^ Marked studies were conducted within adolescent samples [45, 46] or predicted inpatient costs instead of LOS, which are closely associated [35]

Thus, the aim of the present study was to exploratory analyze predictors of LOS within the existing routine care for adult inpatients with AN in Germany and to compare these findings with the clinical theory and the existing evidence.

## Methods

### Design and participants

The present investigation is part of an observational prospective multi-center study on the course of patients with AN during inpatient treatment. Three hospitals (Schoen clinics: Bad Bramstedt, BB; Prien, P; and Hamburg-Eilbek, HH) participated in the study. Recruitment occurred in accordance with the following inclusion criteria: AN as the primary diagnosis, female gender, minimum age of 16 years, and the ability to speak German with adequate fluency. The exclusion criteria were as follows: acute abuse of drugs or alcohol, acute suicidality, the existence of a psychotic or bipolar disorder, or a severe life-threatening somatic disorder. Diagnoses were validated using the German version of the Structured Clinical Interview for DSM-IV Axis I Disorders [22].

Shortly after hospital admission, eligible patients were offered to participate in the study by a psychologist or physician of the respective study center. Prior to inclusion into the study, all participants provided written informed consent. The local research ethics committee approved the study (Medical Chamber Hamburg MC-419/10, the medical association Schleswig-Holstein: AZ 030/10; and the university medical center Munich: 246-10).

### Inpatient treatment program

All three participating hospitals belong to the same hospital network. They share best practice recommendations for the treatment of AN, which were jointly developed. The eating disorder (ED) specialist inpatient units comprise multidisciplinary teams. The therapeutic approach taken by all units is predominantly cognitive behavioral. The therapeutic elements consist of nutritional counselling, eating diaries, meal supervision and support, ED group therapy, psycho-education, emotional competency training, relaxation, art and music therapy, individual psychotherapy and weight contracts defining a target weekly weight gain between 600 and 1000 g. The discharge decision was based on clinical judgement rather than achieving specific cutoff criteria.^1^ However, the best practice manual recommends the achievement of the premorbid or set point weight. If the premorbid weight cannot be determined (i.e. illness onset in adolescence) the target discharge BMI should be ideally 18.5 kg/m^2^ and at least 16 kg/m^2^. Other goals of the ED inpatient treatment include the normalization of ED related behaviors and cognitions, the improvement of somatic symptoms as well as familial and social problems. The costs of the treatment are usually covered fully by private or statutory health insurances.

### Assessment

At admission and discharge, participants completed the standardized questionnaires described in detail below. At admission only, socio-demographic and clinical data (age, age of onset, partner, living situation, marital status, educational level, and number of previous hospitalizations due to mental disorders) were obtained.

#### Body mass index

Patient weight was measured at admission, discharge and at weekly time points during the inpatient stay by a staff member. Height was determined at admission to the nearest 0.1 cm. Using this information, we calculated the body mass index (BMI) as weight (kg)/height (m^2).

#### EDI-2

The German long form of the Eating Disorder Inventory – 2 (EDI-2) consists of 11 subscales: drive for thinness, bulimia, body dissatisfaction, ineffectiveness, perfectionism, interpersonal distrust, interoceptive awareness, maturity fears, asceticism, impulse regulation, and social insecurity. The EDI-2 is a self-assessment rating scale and assesses eating disorder pathology as well as relevant maintaining and etiological factors [23].

#### PHQ-9

The widely used 9-item depression module of the Patient Health Questionnaire (PHQ-9) was completed at admission and discharge, and the sum scores were calculated. The items assess depressive symptoms, and the responses are rated on a 4-point scale that ranges from 0 (never) to 3 (almost every day). The validity and sensitivity to change of the PHQ-9 have been demonstrated in multiple studies [24–26].

#### GAD-7

The German version of the 7-item anxiety module of the Patient Health Questionnaire, the Generalized Anxiety Disorder Scale (GAD-7), was used in this study [26–28]. The items of this questionnaire assess anxiety symptoms, and the responses are rated on a 4-point scale that ranges from 0 (never) to 3 (almost every day).

#### PHQ-15

To assess somatic symptoms, the German version of the 15-item somatic symptoms scale of the Patient Health Questionnaire (PHQ-15) was used [26, 29]. The responses are rated on a 4-point scale that ranges from 0 (never) to 3 (almost every day).

#### Fever

To assess therapy motivation, we used an abbreviated version of the German University of Rhode Island Change Assessment Scale. The validity of the instrument was tested within a sample of patients with eating disorders and was found to be good [30]. We calculated a sum score.

### Statistical analysis

Descriptive statistics were calculated separately for therapy completers and non-completers. Two-tailed t-tests and chi-square tests were conducted to determine differences between therapy completers and non-completers.

To predict the LOS, a stepwise linear regression analysis was performed. Pre-analyses were conducted to determine whether the variables fulfilled the assumptions required by the regression analysis. The variables age, FEVER sum score and the EDI-2 subscale score on bulimia were highly skewed. We could not reach normal distributions using common transformations (log, exponential, square root) and hence decided to dichotomize these variables using a median split. All categorical and ordinal variables were dummy-coded [31, 32]. In detail, we included the following predictors: median-split age (24 years and younger; 25 years and older), mean-centered age of onset, living alone (yes; no), partner (yes; no), education (base level: primary education; contrasts: secondary education, university degree, other), previous hospitalizations due to a mental disorder (yes; no), AN subtype (restrictive; purging), mean-centered admission BMI, dummy variables for the centers (base level: BB; contrasts: HH or P), admission PHQ-9 score, admission PHQ-15 score, admission GAD-7 score, median-split FEVER (sum score of 42 and below vs. above 42), and all EDI-2 subscales. In light of the conflicting evidence base and a large number of potentially relevant predictor variables we conducted an exploratory analysis using a stepwise variable selection procedure. The stepwise selection added terms with *p* < .05 and removed those with *p* > .10. The prediction of LOS was conducted solely within the sample of therapy completers. This was done to avoid confounding predictors of therapy duration with predictors of therapy completion, which comprise a separate research topic. Missing data within predictor variables also reduce the analyzed sample size. As a sensitivity analysis we computed a model using multiple imputations by chained equations to fill missing data within the predictor variables. The results of this sensitivity analysis are reported in relation to the results of the unimputed model.

All analyses were conducted using Stata 13 SE.

## Results

### Flow of study participants

Participants were enrolled during a period of 27 months between April 2010 and July 2012. We recruited 233 patients. Twenty-five patients were removed from the analysis due to implausible or missing data (e.g. missing admission BMI). Of the 208 remaining patients, 176 patients completed the therapy, which indicates a non-completion rate of 15.4%. For the present study, data from these 176 treatment-completers were analyzed. Stepwise regression analyses included patients with complete data on all predictor variables, which resulted in sample sizes of 135 participants.

### Sample characteristics

Descriptive information regarding demographic and clinical characteristics of the sample are summarized in Table 2. The study sample consisted of patients with a mean age of 27.1 years (SD = 8.9) and an average LOS of 11.8 weeks (SD = 5.2). Separated by treatment center the average LOS was 11.7 weeks (SD = 5.1) in center BB, 7.5 weeks (SD = 2.2) in center HH, and 14.4 weeks (SD = 5.1) in center P. The patients of the different treatments centers do not differ significantly with respect to sociodemographic characteristics (mean age center BB = 26.6 years, SD = 9.4; mean age center HH = 27.7 years, SD = 8.6; mean age center *P* = 28.2 years, SD = 8.2; F = 0.54; *p* = .58) or baseline BMI (mean BMI t0 center BB = 15.1 kg/m^2^, SD = 1.5; mean BMI t0 center HH = 15.0 kg/m^2^, SD = 1.8; mean BMI t0 center *P* = 14.9 kg/m^2^, SD = 1.8; F = 0.42; *p* = .66). The BMI significantly increased from admission (mean = 15.0 kg/m^2^, SD = 1.6) to discharge (mean = 17.1 kg/m^2^, SD = 1.5; *t* = −20.1, *p* < .001). Therapy completers remained in inpatient treatment significantly longer than therapy non-completers (11.8 weeks, SD = 5.2 vs. 5.1 weeks, SD = 3.1; *t* = 7.01, p < .001). There were no significant differences between treatment completers and non-completers with respect to any other demographic or clinical variables.

**Table 2:** Demographic and clinical characteristics

Sample (*n* = 176)	
Age M (SD)	27.1 (8.9)
Admission BMI M (SD)	15.0 (1.6)
AN subtype^a^ N (%)	
Restricting	88 (54.0)
Purging	75 (46.0)
Age of onset^a^ M (SD)	17.8 (6.0)
Previous inpatient treatment^a^ N (%)	
Yes	102 (60.0)
No	68 (40.0)
Education^a^ N (%)	
Primary Education	53 (30.8)
Secondary Education	74 (43.0)
University Degree	22 (12.8)
Student and other	23 (13.4)
Marital Status^a^ N (%)	
Single	143 (83.1)
Married	21 (12.2)
Divorced and widowed	8 (4.7)
Partnership^a^ N (%)	
Yes	57 (32.9)
No	116 (67.1)
Center N (%)	
HH	29 (16.5)
P	46 (26.1)
BB	101 (57.4)

^a^ Variable contains missing values. The percentages are calculated based on all patients with non-missing information on the respective variable

### Predictors of length of stay (LOS)

A lower admission BMI (b = −1.66, *p* < .001), the purging AN-subtype (b = 1.91, *p* = .013) and higher values on the EDI-2 asceticism subscale (b = 0.12, *p* = .030) predicted significantly longer treatment durations (see Table 3). We also identified significant differences among the three participating hospitals. Study center HH was associated with shorter inpatient treatment durations, and study center P was associated with longer inpatient treatment durations, when compared to the third center, BB. No other variable significantly contributed to the prediction of LOS. The predictors explained 36.5% of the variance (Adj. R^2^ = .365).

**Table 3:** Stepwise linear regression analysis on the length of stay in AN inpatients (*N* = 135)

Dependent Variable	Predictors	b	95% CI	SE	t	*p*	F	*p*	Adj. R^2^
Length of Stay	Admission BMI	−1.66	[−2.15, −1.18]	.245	−6.78	<.001	16.39	<.001	.365
	Center HH	−4.91	[−7.73, −2.09]	1.427	−3.44	.001			
	Center P	2.34	[0.70, 3.98]	.827	2.83	.005			
	Purging subtype	1.91	[0.41, 3.40]	.754	2.53	.013			
	Asceticism	0.12	[0.01, 0.23]	.054	2.19	.030			

*CI* Confidence interval, *SE* Standard error, center *HH* Hamburg Eilbek, center *P* Prien. The base level of the categorical variable center was: Bad Bramstedt (BB). Length of stay (LOS) is specified in weeks. The included non-influential variables were age, age of onset, living situation, partnership status, education (base level: primary education), previous hospitalization, self-rated depression, anxiety and somatic symptoms (PHQ-9, PHQ-15, GAD-7), self-rated therapy motivation (FEVER) and the EDI-2 subscale scores on drive for thinness, bulimia, body dissatisfaction, ineffectiveness, perfectionism, interpersonal distrust, interoceptive awareness, maturity fears, impulse regulation, and social insecurity

In the sensitivity analysis using the imputed data all significant predictor variables were confirmed except of the EDI-2 asceticism subscale (admission BMI: b = −1.50, *p* < .001; center HH: b = −4.19, p < .001; center P: b = 2.62, *p* = .001; purging subtype: b = 1.43, *p* = .03). Instead the EDI-2 subscale ineffectiveness (b = 0.07, *p* = .05) reached the significance level.

## Discussion

The present analysis found that patients with a longer hospital stay were characterized by a lower admission BMI, the purging AN-subtype and higher values on the EDI-2 asceticism subscale. The findings seem clinically plausible, as these patients represent more severely affected subgroups. Furthermore, marked differences between treatment sites emerged.

So far, the evidence base on predictors of LOS is highly inconsistent (s. Table.1). This might not be surprising considering the fact that LOS is influenced by the respective health care system and the studies stem from various different time periods and locations. However, predictors which can be reliably replicated might be clinically meaningful irrespective of system level variables or current health care policies [33]. Thus, BMI at admission emerged as a significant predictor consistently within several studies [34, 35], with only one exception [36]. Whereas the age of the patient significantly predicted LOS only in one study from 1995 and other investigations like the present one could not replicate this result [35]. Surprisingly, AN-subtype has not been considered in previous studies as a potential predictor of LOS. However, a similar result to the present one has been demonstrated by a recent study investigating patients with extreme underweight. Here, patients with the purging AN-subtype showed less weight gain per week and significantly longer inpatient treatment durations [37]. Another study found that the binge/purge subtype significantly predicted higher cost in AN outpatients [38]. Regarding patients with higher asceticism values, we note that no previous studies investigating LOS in AN patients included self-rated psychopathology as a potential predictor. We found that asceticism is associated with LOS and hypothesize that AN symptoms might be more ego-syntonic for patients with higher levels of asceticism [23]. Thus, these patients might require additional time to achieve changes. However, the importance of the subscale is not definite since it was not confirmed within the sensitivity analysis. Instead the results of the imputed model indicated that patients with more pronounced ineffectiveness scores stay longer.

Moreover, marked differences between treatment sites emerged. Based on the existing evidence these differences were not to be expected. To our knowledge, only one study [16] points to large differences in LOS between different eating disorder specialist units. However, this study investigated AN treatment centers all across Europe. Another study found no differences in LOS between four treatment sites within the context of the same national health care system (Australia). Moreover, compared to a fifth treatment site in another country (New Zealand) a significant difference was found only in the univariate analysis (s. Table 1) [34]. Presently, the analyzed treatment sites are not only located in the same health care system but also belong to the same hospital network and share best practice recommendations. Against this background, the found differences highlight the importance of analyzing geographic variations in mental health care [39]. Such variations might be associated with the population size and the supply of other mental health services in the surrounding region [34]. Accordingly, the shortest LOS was identified for the hospital located in an urban region, where it may be easier to find suitable follow-up therapy. The longest stays were identified within a hospital where it is more common to treat patients who do not live nearby and therefore arranging follow-up therapy may be more challenging.

Moreover, it seems noteworthy that the variable ‘previous hospitalizations’ did not emerge as a significant predictor of LOS. The finding that a longer LOS of the first admission reduces long-term mortality [15], might imply extended durations for the initial treatment. However, we did not find such an effect in the presently analyzed sample.

Finally, as an auxiliary finding we noticed a mean discharge BMI (17.1 kg/m^2^, SD = 1.5) that is below the diagnostic cut-off of 17.5 kg/m^2^ as specified by the International Classification of Diseases (ICD-10) [40]. On the one hand, this finding is troublesome since the discharge BMI is associated with the risk of readmission [9, 10]. On the other hand, a discharge BMI below the diagnostic cut-off is unfortunately not uncommon. Two other relatively recent studies on adult AN inpatient treatments report similar discharge BMIs of 17.2 kg/m^2^ (SD = 1.9) and 17.3 kg/m^2^ (2.1), respectively [41, 42]. Further research on the interrelatedness of the discharge BMI, the length of stay, other patient and health care system related factors and the long-term outcome in naturalistic settings seems important.

The strengths of our study include the large sample size, the prospective multi-center design and the broad list of included potential predictors, such as eating disorder- and non-eating disorder-related psychological variables. By comparing the present results from our German sample with existing international findings we could gain insights into stable, location-independent and clinically meaningful predictors of LOS in patients with AN. Based on the findings of the predictors we cannot finally answer the important question on the optimal LOS for a certain patient, yet we can inform future studies on this topic. On the one hand, a limitation of this study is that we could not consider all variables with a potential influence on treatment duration (e.g., duration of untreated illness, weight suppression, availability of alternative treatment options). On the other hand, the number of included predictors was quite high relative to the sample size. Based on the existing evidence a more stringent theoretical selection of predictor variables was not possible. However, the applied variable selection procedure can lead to instable models. This relatively common problem of prediction research further adds to the importance of replication [33]. Another limitation of the present study concerns the number of patients that could not be included in the final analysis Whereas the treatment non-completion rate was well within an usual range [43], the missing or implausible admission information for several patients due to data collection problems in the beginning of the study could have been avoidable.

## Conclusions

To date, decisions on LOS in patients with AN are not guided by evidence or standardized. The results of the present investigation into factors that influence LOS could contribute to the inconsistent evidence base on the subject. Admission BMI and to a lesser degree AN-subtype emerged in this study as well in previous international studies as factors significantly associated with LOS in patients with AN. These observed factors that influence the current clinical decision-making seem clinically plausible and they appear to be meaningful irrespective of the specific health care system. Both factors are routinely assessed at admission and might be considered within health care policies, specifically within reimbursement schemes. A standardization of the reimbursement, and consequently, the LOS based on lump sums over diagnostic groups disregards these factors [44]. A more differentiated approach that, at a minimum, considers BMI at admission and AN-subtype seems to be recommendable. The found differences between the treatment sites highlight that it is important to discuss the standards for determining the length of stay. Future research on geographical variations in mental health care seems recommended.

## Abbreviations

AN: Anorexia nervosa
BB: Center Bad Bramstedt
BMI: Body-Mass-Index
CI: Confidence interval
ED: Eating disorders
EDI: Eating Disorder Inventory
GAD-7: Generalized Anxiety Disorder Scale
HH: Center Hamburg Eilbek
LOS: Length of stay
P: Center Prien
PHQ: Patient Health Questionnaire
SE: Standard error

## Footnotes

1 In the presence of a definite target weight the length of stay would be a measure of treatment response, which is presently not the case. The focus is instead on the clinical decision making.
